# Supplementary material for: Sexual dimorphism in skull size and shape of Laticauda colubrina (Serpentes: Elapidae)
Source: PeerJ. 2023 Oct 18;11:e16266. doi: 10.7717/peerj.16266 (PMC10590095; doi:10.7717/peerj.16266)
Supplement: Supplemental Information 1 — All specimens are from the collection of Field Museum of Natural History, Chicago. [file peerj-11-16266-s001.docx]

List of specimens of *Laticauda colubrina* used in present study. All specimens are from the collection of the Field Museum of Natural History, Chicago.

Females: FMNH 236244, 236254, 236550, 236249, 236247, 236246, 236240, 236237, 235390, 235389, 236239, 236336, 235407, 236241, 235387, 236235, 236233, 235398, 236234, 236256, 236257, 234154, 236269, 236268, 234153, 236559, 236260, 236270, 234150, 236265, 236264, 236258, 236272, 235415; Males: FMNH 236318, 236302, 236293, 236316, 236314, 236324, 236323, 235410, 236322, 235400, 235402, 235388, 235425, 235417, 235399, 236315, 236307, 135412, 234157, 234165, 234160, 236326, 236325, 234152, 234158, 234163, 234151
