# Supplementary material for: Sexual dimorphism in skull size and shape of Laticauda colubrina (Serpentes: Elapidae)
Source: PeerJ. 2023 Oct 18;11:e16266. doi: 10.7717/peerj.16266 (PMC10590095; doi:10.7717/peerj.16266)
Supplement: Supplemental Information 3 [file peerj-11-16266-s003.docx]

|  | PC1 | PC2 | PC3 | PC4 | PC5 | PC6 | PC7 | PC8 |
| --- | --- | --- | --- | --- | --- | --- | --- | --- |
| SL | **-0.812** | 0.255 | -0.206 | 0.195 | 0.053 | 0.006 | -0.131 | -0.033 |
| SH | -0.619 | 0.283 | -0.278 | -0.001 | -0.016 | -0.067 | -0.169 | -0.073 |
| SW | 0.019 | 0.080 | -0.100 | 0.199 | -0.366 | -0.594 | 0.140 | 0.126 |
| PW1 | **-0.859** | 0.123 | -0.013 | -0.120 | 0.053 | 0.070 | 0.120 | -0.129 |
| PW2 | **-0.959** | 0.136 | -0.098 | 0.075 | 0.047 | -0.025 | -0.090 | -0.091 |
| PAR | -0.386 | 0.250 | -0.101 | 0.230 | 0.173 | -0.255 | -0.274 | -0.022 |
| NCL | 0.430 | 0.129 | 0.080 | -0.044 | 0.258 | -0.459 | 0.097 | -0.163 |
| NL | -0.220 | -0.034 | -0.478 | -0.714 | 0.072 | -0.018 | 0.161 | 0.239 |
| NW | 0.369 | 0.078 | 0.140 | -0.246 | 0.146 | 0.113 | 0.497 | -0.293 |
| FL | **-0.736** | 0.081 | 0.256 | -0.236 | 0.170 | -0.134 | -0.109 | 0.218 |
| FW1 | -0.026 | -0.157 | 0.550 | -0.128 | -0.323 | 0.422 | -0.367 | 0.162 |
| FW2 | -0.690 | -0.090 | 0.121 | -0.087 | -0.448 | 0.041 | 0.140 | -0.155 |
| PLL | 0.290 | 0.128 | 0.026 | 0.199 | -0.381 | -0.103 | -0.257 | 0.294 |
| PTL | 0.302 | 0.216 | -0.317 | 0.258 | -0.066 | 0.181 | 0.174 | 0.010 |
| PTTL | 0.205 | 0.307 | -0.055 | 0.467 | 0.000 | 0.353 | -0.007 | -0.431 |
| PMW | 0.443 | 0.044 | 0.404 | -0.340 | 0.272 | 0.412 | 0.166 | 0.005 |
| PRETR | 0.541 | 0.053 | -0.347 | 0.405 | -0.155 | 0.258 | 0.066 | -0.082 |
| PML | 0.262 | 0.094 | 0.638 | 0.255 | 0.366 | -0.344 | 0.129 | 0.032 |
| MXL | 0.331 | -0.071 | 0.352 | -0.180 | 0.032 | 0.001 | -0.109 | 0.128 |
| MDL | 0.266 | -0.021 | -0.025 | 0.247 | 0.050 | -0.113 | 0.007 | 0.027 |
| MD2L | **0.715** | 0.183 | -0.042 | 0.219 | 0.172 | -0.110 | 0.045 | 0.098 |
| DENT | 0.096 | 0.116 | 0.505 | -0.036 | -0.108 | 0.050 | -0.265 | 0.116 |
| FMDB | 0.557 | 0.211 | -0.490 | 0.251 | 0.173 | 0.025 | -0.189 | 0.228 |
| ECT | 0.407 | -0.140 | -0.237 | -0.068 | 0.524 | 0.301 | -0.233 | 0.118 |
| QL | **0.722** | 0.028 | -0.058 | -0.444 | -0.173 | -0.218 | -0.233 | -0.329 |
| CQL | -0.210 | -0.071 | 0.052 | 0.106 | -0.374 | 0.193 | 0.551 | 0.335 |
| PFL | -0.153 | **-0.963** | -0.088 | 0.146 | 0.061 | -0.077 | -0.030 | -0.055 |
| PFH | 0.100 | -0.266 | 0.022 | -0.197 | 0.256 | 0.360 | 0.263 | -0.305 |
| STP | **0.798** | -0.012 | -0.068 | 0.091 | -0.238 | -0.089 | 0.088 | 0.177 |
| % variation | 33.68 | 10.21 | 7.88 | 7.65 | 5.28 | 5.06 | 3.85 | 3.62 |
