# Supplementary material for: Sexual dimorphism in skull size and shape of Laticauda colubrina (Serpentes: Elapidae)
Source: PeerJ. 2023 Oct 18;11:e16266. doi: 10.7717/peerj.16266 (PMC10590095; doi:10.7717/peerj.16266)
Supplement: Supplemental Information 4 — The distances that show positive allometry are highlighted red and those that show negative allometry are highlighted blue. Asterix denotes p-value less than *<0.05, **<0.01, ***<0.001. [file peerj-11-16266-s004.docx]

| Baseline | Character | Sex | Intercept | Intercept 95%  Confidence Intervals | | Slope | | Slope 95%  Confidence Intervals | | R^2^ | Sig. |
| --- | --- | --- | --- | --- | --- | --- | --- | --- | --- | --- | --- |
| SVL | BW | F | -8.316 | -9.897 | -6.735 | 3.638 | 3.115 | | 4.161 | 0.92 | ** |
|  |  | M | -8.769 | -10.72 | 6.918 | 3.829 | 3.154 | | 4.505 | 0.9 | ** |
|  | SL | F | -1.367 | -1.828 | -9.069 | 0.896 | 0.743 | | 1.049 | 0.88 | n.s. |
|  |  | M | -1.433 | -1.986 | -0.88 | 0.926 | 0.735 | | 1.118 | 0.85 | n.s. |
|  | SH | F | -1.955 | -2.46 | -1.45 | 0.914 | 0.746 | | 1.081 | 0.86 | n.s. |
|  |  | M | -1.795 | -2.369 | -1.22 | 0.865 | 0.666 | | 1.064 | 0.812 | n.s. |
|  | SW | F | -2.115 | -2.825 | -1.406 | 1.059 | 0.823 | | 1.294 | 0.78 | n.s. |
|  |  | M | -2.392 | -3.135 | -1.65 | 1.162 | 0.905 | | 1.419 | 0.826 | n.s. |
|  | PW1 | F | -1.783 | -2.199 | -1.367 | 0.897 | 0.759 | | 1.035 | 0.903 | n.s. |
|  |  | M | -1.72 | -2.333 | -1.108 | 0.886 | 0.674 | | 1.098 | 0.796 | n.s. |
|  | PW2 | F | -0.4741 | -0.701 | -0.247 | 0.427 | 0.352 | | 0.503 | 0.873 | *** |
|  |  | M | -1.287 | -1.704 | -0.871 | 0.716 | 0.572 | | 0.86 | 0.856 | *** |
|  | PAR | F | -1.799 | -2.25 | -1.347 | 0.929 | 0.779 | | 1.079 | 0.984 | n.s. |
|  |  | M | -1.903 | -2.613 | -1.193 | 0.969 | 0.723 | | 1.214 | 0.771 | n.s. |
|  | NCL | F | -2.457 | -3.05 | -1.864 | 1.083 | 0.886 | | 1.279 | 0.865 | n.s. |
|  |  | M | -2.841 | -3.735 | -1.946 | 1.217 | 0.907 | | 1.526 | 0.77 | n.s. |
|  | NL | F | -2.388 | -2.975 | -1.801 | 0.952 | 0.757 | | 1.146 | 0.829 | n.s. |
|  |  | M | -2.463 | -3.484 | -1.441 | 0.979 | 0.626 | | 1.332 | 0.536 | n.s. |
|  | NW | F | -3.342 | -3.948 | -2.736 | 1.301 | 1.1 | | 1.502 | 0.902 | ** |
|  |  | M | -2.744 | -3.849 | -1.638 | 1.108 | 0.725 | | 1.49 | 0.576 | n.s. |
|  | FL | F | -1.429 | -2.088 | -0.77 | 0.628 | 0.409 | | 0.846 | 0.505 | ** |
|  |  | M | -2.916 | -3.801 | -2.032 | 1.184 | 0.878 | | 1.49 | 0.762 | n.s. |
|  | FW1 | F | -2.765 | -3.228 | -2.301 | 1.187 | 1.033 | | 1.341 | 0.932 | ** |
|  |  | M | -3.182 | -4.146 | -2.218 | 1.347 | 1.014 | | 1.681 | 0.782 | * |
|  | FW2 | F | -2.093 | -2.526 | -1.66 | 0.927 | 0.783 | | 1.07 | 0.902 | n.s. |
|  |  | M | -2.614 | -3.634 | -1.594 | 1.125 | 0.772 | | 1.478 | 0.65 | n.s. |
|  | PLL | F | -2.625 | -3.223 | -2.028 | 1.107 | 0.908 | | 1.305 | 0.869 | n.s. |
|  |  | M | -3.427 | -4.481 | -2.374 | 1.389 | 1.025 | | 1.754 | 0.755 | * |
|  | PTL | F | -2.272 | -2.94 | -1.604 | 1.172 | 0.951 | | 1.394 | 0.854 | n.s. |
|  |  | M | -1.876 | -.2504 | -1.248 | 1.043 | 0.826 | | 1.26 | 0.845 | n.s. |
|  | PTTL | F | -2.307 | -2.905 | -1.71 | 1.129 | 0.931 | | 1.328 | 0.874 | n.s. |
|  |  | M | -2.707 | -3.78 | -1.634 | 1.279 | 0.907 | | 1.65 | 0.7 | n.s. |
|  | PMW | F | -3.223 | -3.725 | -2.72 | 1.274 | 1.107 | | 1.44 | 0.93 | ** |
|  |  | M | -3.703 | -4.656 | -2.75 | 1.45 | 1.121 | | 1.78 | 0.816 | ** |
|  | PRETR | F | -3.081 | -4.119 | -2.044 | 1.167 | 0.823 | | 1.511 | 0.645 | n.s. |
|  |  | M | -2.673 | -3.532 | -1.813 | 1.027 | 0.73 | | 1.325 | 0.702 | n.s. |
|  | MXL | F | -2.706 | -3.24 | -2.172 | 1.114 | 0.937 | | 1.292 | 0.897 | n.s. |
|  |  | M | -3.711 | 04.595 | -2.828 | 1.471 | 1.165 | | 1.776 | 0.846 | ** |
|  | MDL | F | -1.897 | -2.587 | -1.206 | 1.102 | 0.973 | | 1.331 | 0.824 | ** |
|  |  | M | -1.959 | -2.613 | -1.305 | 1.131 | 0.905 | | 1.358 | 0.858 | n.s. |
|  | MD2L | F | -1.9 | -2.438 | -1.363 | 1.086 | 0.908 | | 1.265 | 0.89 | n.s. |
|  |  | M | -2.144 | -2.827 | -1.462 | 1.174 | 0.938 | | 1.41 | 0.856 | n.s. |
|  | DENT | F | -2.259 | -2.809 | -1.71 | 1.048 | 0.865 | | 1.23 | 0.877 | n.s. |
|  |  | M | -3.207 | -4.061 | -2.353 | 1.382 | 1.087 | | 1.678 | 0.837 | ** |
|  | FMDB | F | -2.598 | -3.254 | -1.941 | 1.137 | 0,919 | | 1.354 | 0.850 | n.s. |
|  |  | M | -2.729 | -3.747 | -1.71 | 1.18 | 0.827 | | 1.532 | 0.682 | n.s. |
|  | ECT | F | -2.331 | -2.842 | -1.82 | 1.112 | 0.942 | | 1.281 | 0.905 | n.s. |
|  |  | M | -2.906 | -3.976 | -1.837 | 1.312 | 0.942 | | 1.682 | 0.717 | * |
|  | QL | F | -3.402 | -4.469 | -2.334 | 1.405 | 1.05 | | 1.759 | 0.741 | * |
|  |  | M | -3.12 | -3.846 | -2.394 | 1.318 | 1.067 | | 1.57 | 0.871 | ** |
|  | CQL | F | -2.935 | -3.453 | -2.418 | 1.17 | 0.998 | | 1.342 | 0.912 | * |
|  |  | M | -3.027 | -3.875 | -2.178 | 1.219 | 0.925 | | 1.512 | 0.793 | n.s. |
|  | PFH | F | -2.296 | -3.101 | -1.491 | 1.01 | 0.743 | | 1.277 | 0.715 | n.s. |
|  |  | M | -2.656 | -3.598 | -1.714 | 1.135 | 0.809 | | 1.461 | 0.706 | n.s. |
|  | STP | F | -2.947 | -3.612 | -2.281 | 1.298 | 1.077 | | 1.519 | 0.882 | ** |
|  |  | M | -3.013 | -3.975 | -2.05 | 1.325 | 0.992 | | 1.658 | 0.775 | * |
