# Supplementary material for: Sexual dimorphism in skull size and shape of Laticauda colubrina (Serpentes: Elapidae)
Source: PeerJ. 2023 Oct 18;11:e16266. doi: 10.7717/peerj.16266 (PMC10590095; doi:10.7717/peerj.16266)
Supplement: Supplemental Information 5 — The distances that show positive allometry are highlighted red and those that show negative allometry are highlighted blue. Asterix denotes p-value less than *<0.05, **<0.01, ***<0.001. [file peerj-11-16266-s005.docx]

| **Baseline** | **Character** | **Sex** | **Intercept** | **Intercept 95%**  **Confidence Intervals** | | **Slope** | **Slope 95%**  **Confidence Intervals** | | **R^2^** | **Sig.** |
| --- | --- | --- | --- | --- | --- | --- | --- | --- | --- | --- |
| SL | SH | F | -0.631 | -0.718 | -0.573 | 1.075 | 1.03 | 1.138 | 0.982 | ** |
|  |  | M | -0.512 | -0.625 | -0.398 | 0.98 | 0.889 | 1.07 | 0.95 | n.s. |
|  | SW | F | -0.5459 | -0.7095 | -0.3823 | 1.217 | 1.096 | 1.338 | 0.924 | *** |
|  |  | M | -0.577 | -0.909 | -0.246 | 1.238 | 0.973 | 1.503 | 0.73 | * |
|  | PW1 | F | -0.409 | -0.553 | -0.266 | 0.998 | 0.891 | 1.105 | 0.912 | n.s. |
|  |  | M | -0.436 | -0.618 | -0.255 | 1.03 | 0.885 | 1.175 | 0.883 | n.s. |
|  | PW2 | F | 0.125 | 8.138e-3 | 0.242 | 0.516 | 0.429 | 0.603 | 0.783 | *** |
|  |  | M | -0.167 | -0.323 | -0.011 | 0.762 | 0.638 | 0.887 | 0.843 | *** |
|  | PAR | F | -0.418 | -0.516 | -0.32 | 1.063 | 0.991 | 1.135 | 0.967 | * |
|  |  | M | -0.488 | -0.681 | -0.292 | 1.111 | 0.957 | 1.265 | 0.887 | n.s. |
|  | NCL | F | -0.859 | -0.989 | -0.728 | 1.249 | 1.152 | 1.346 | 0.954 | *** |
|  |  | M | -0.962 | -1.228 | -0.695 | 1.318 | 1.105 | 1.531 | 0.846 | ** |
|  | NL | F | -1.093 | -1.337 | -0.849 | 1.18 | 0.999 | 1.361 | 0.819 | * |
|  |  | M | -1.019 | -1.377 | -0.661 | 1.118 | 0.832 | 1.404 | 0.614 | n.s. |
|  | NW | F | -1.311 | -1.455 | -1.166 | 1.419 | 1.312 | 1.527 | 0.956 | *** |
|  |  | M | -1.165 | -1.503 | -0.828 | 1.311 | 1.042 | 1.581 | 0.762 | * |
|  | FL | F | -1.05 | -1.459 | -0.641 | 1.165 | 0.8616 | 1.469 | 0.496 | n.s. |
|  |  | M | -1.086 | -1.354 | -0.817 | 1.283 | 1.068 | 1.498 | 0.835 | ** |
|  | FW1 | F | -1.067 | -1.355 | -0.779 | 1.408 | 1.195 | 1.622 | 0.824 | *** |
|  |  | M | -1.105 | -1.258 | -0.952 | 1.462 | 1.34 | 1.584 | 0.959 | *** |
|  | FW2 | F | -0.775 | -1.000 | -0.551 | 1.115 | 0.948 | 1.282 | 0.828 | n.s. |
|  |  | M | -0.937 | -1.175 | 0.699 | 1.265 | 1.075 | 1.455 | 0.867 | * |
|  | PLL | F | -0.927 | -1.04 | 0.814 | 1.23 | 1.146 | 1.314 | 0.964 | *** |
|  |  | M | -1.312 | -1.614 | -1.01 | 1.528 | 1.287 | 1.769 | 0.853 | *** |
|  | PTL | F | -0.448 | -0.626 | -0.271 | 1.28 | 1.148 | 1.411 | 0.919 | *** |
|  |  | M | -0.282 | -0.417 | -0.146 | 1.142 | 1.034 | 1.25 | 0.947 | ** |
|  | PTTL | F | -0.307 | -0.504 | -0.11 | 1.063 | 0.971 | 1.21 | 0.855 | n.s. |
|  |  | M | -0.741 | -1.01 | -0.381 | 1.388 | 1.1 | 1.676 | 0.747 | ** |
|  | PMW | F | -1.235 | -1.352 | -1.118 | 1.387 | 1.3 | 1.474 | 0.97 | *** |
|  |  | M | -1.473 | -1.797 | 1.149 | 1.583 | 1.324 | 1.842 | 0.843 | *** |
|  | PRETR | F | -1.311 | -1.571 | -1.052 | 1.311 | 1.118 | 1.504 | 0.834 | ** |
|  |  | M | -1.25 | -1.529 | -0.971 | 1.246 | 1.023 | 1.469 | 0.812 | * |
|  | MXL | F | -1.028 | -1.107 | -0.95 | 1.266 | 1.208 | 1.325 | 0.984 | *** |
|  |  | M | -1.416 | -1.639 | -1.194 | 1.573 | 1.395 | 1.75 | 0.925 | *** |
|  | MDL | F | -0.236 | -0.409 | -0.063 | 1.249 | 1.121 | 1.378 | 0.919 | *** |
|  |  | M | -0.263 | -0.385 | -0.141 | 1.267 | 1.169 | 1.365 | 0.965 | *** |
|  | MD2L | F | -0.259 | -0.329 | -0.186 | 1.226 | 1.173 | 1.278 | 0.986 | *** |
|  |  | M | -0.372 | -0.847 | -0.257 | 1.304 | 1.212 | 1.396 | 0.971 | *** |
|  | DENT | F | -0.698 | -0.8 | -0.596 | 1.2 | 1.125 | 1.276 | 0.97 | *** |
|  |  | M | -1.032 | -1.201 | -0.863 | 1.464 | 1.329 | 1.599 | 0.95 | *** |
|  | FMDB | F | -0.944 | -1.135 | -0.753 | 1.323 | 1.182 | 1.465 | 0.912 | *** |
|  |  | M | -0.971 | -1.3 | -0.642 | 1.327 | 1.064 | 1.59 | 0.769 | ** |
|  | ECT | F | -0.617 | -0.718 | -0.516 | 1.228 | 1.153 | 1.303 | 0.971 | *** |
|  |  | M | -0.996 | -1.379 | -0.612 | 1.507 | 1.201 | 1.813 | 0.757 | *** |
|  | QL | F | -1.331 | -1.614 | -1.048 | 1.628 | 1.418 | 1.838 | 0.873 | *** |
|  |  | M | -1.091 | -1.312 | -0.869 | 1.277 | 1.1 | 1.454 | 0.887 | ** |
|  | CQL | F | -1.165 | -1.338 | -0.992 | 1.314 | 1.185 | 1.443 | 0.927 | *** |
|  |  | M | -1.091 | -1.312 | -0.869 | 1.277 | 1.1 | 1.454 | 0.887 | ** |
|  | PFH | M | -0.773 | -0.953 | -0.594 | 1.144 | 1.011 | 1.277 | 0.896 | * |
|  |  | F | -1.037 | -1.318 | -0.756 | 1.343 | 1.119 | 1.568 | 0.836 | ** |
|  | STP | M | -1.023 | -1.161 | -0.885 | 1.489 | 1.386 | 1.591 | 0.965 | *** |
|  |  | M | -1.007 | -1.235 | -0.779 | 1.465 | 1.282 | 1.647 | 0.908 | *** |
